# GERD after Roux-en-Y Gastric Bypass: Prevalence and Risk Factors Analysis

**DOI:** 10.3390/medicina60081221

**Published:** 2024-07-28

**Authors:** Matas Pažusis, Gabrielė Gerasimovič, Rūta Petereit, Rita Gudaitytė, Almantas Maleckas

**Affiliations:** 1 Department of Surgery, Medical Academy, Lithuanian University of Health Science, 44307 Kaunas, Lithuania; matapazu0229@kmu.lt (M.P.); gabrgera1212@kmu.lt (G.G.); rita.gudaityte2@lsmuni.lt (R.G.); 2 Department of Gastroenterology, Medical Academy, Lithuanian University of Health Science, 44307 Kaunas, Lithuania; ruta.petereit@lsmuni.lt; 3 Department of Gastrosurgical Research and Education, Sahlgrenska Academy, University of Gothenburg, 405 30 Gothenburg, Sweden

**Keywords:** bariatric surgery, Roux-en-Y gastric bypass, GERD, prevalence, risk factors

## Abstract

*Background and Objectives:* The prevalence of GERD is increasing among individuals with obesity, and RYGB is an effective procedure to control GERD and obesity. However, some patients continue to have GERD after RYGB. The aim of this study was to investigate the prevalence and the risk factors for GERD after RYGB. *Material and Methods*: This prospective study included 180 RYGB patients followed for an average of 12.2 (0.6) years. In total, 126 (70%) patients agreed to participate and provided data on their weight, GERD symptoms, and filled the GERD-HRQL, TFEQ-18, and GSRS questionnaires. *Results:* The average age before surgery was 42.7 (10.5) years, and BMI was 45.2 (6.4) kg/m^2^. Moreover, 128 (71.1%) were females, and preoperative GERD was diagnosed in 74 (41.1%) patients. At the 12-year follow-up, the mean %EBMIL and %TWL was 60.37 and 25.73, respectively. The median %WR was 18.0 (39.0). Postoperative GERD was present in 30 (23.8%) patients, of whom 12 (40%) continued to have GERD symptoms and 18 (60%) developed de novo GERD. The GERD-HRQL score significantly decreased from 3.0 (9.0) at baseline to 2.0 (5.0) (*p* = 0.028) at 12 years. GSRS Diarrhea and Indigestion scores increased significantly from 1.33 (0.67) to 1.5 (2.42) (*p* < 0.001) and from 2.0 (1.25) to 2.25 (1.25) (*p* < 0.001), respectively. No change in the cognitive restraint score was observed. Uncontrolled eating and emotional eating scores decreased from 51.85 (22.22) to 40.74 (33.33) (*p* < 0.001) and from 44.44 (44.44) to 33.33 (22.22) (*p* < 0.001), respectively. In the multivariate analysis, %WR > 11 (OR = 3.22, *p* = 0.029) and GSRS Diarrhea score (OR = 3.21, *p* = 0.027) were significant predictors of GERD 12 years after RYGB. *Conclusions:* RYGB was an effective procedure to control GERD; however, 23.8% had persistent or de novo GERD after 12 years. The independent risk factors associated with GERD after RYGB were weight regain and GSRS Diarrhea score.

## 1. Introduction

The prevalence of obesity has steadily increased during the recent five decades. In 2022, 890 million of adults aged 18 years and above were living with obesity [1]. Obesity is a risk factor for cardiovascular, metabolic, musculoskeletal diseases, and some types of cancer and has a direct impact on overall mortality and life expectancy. A recent UK population-based cohort study of 2 million individuals has shown that the all-cause mortality rate among persons with BMI ≥ 25 kg/m^2^ is higher and rises by 21% per each 5 kg/m^2^ increase in BMI units [2]. Furthermore, in a Swedish Obese Subject (SOS) study, after a median 20–22-year follow-up, patients with class II obesity or higher had an 8.5-year shorter adjusted median life expectancy compared to the reference population [3].

There is also a strong association between obesity and gastroesophageal reflux disease (GERD). Hampel H. et al. [4] performed a meta-analysis and found that BMI ≥ 30 kg/m^2^ is associated with a 1.9-fold increase in the risk of GERD symptoms and a 2.8-fold increase in the risk of esophageal adenocarcinoma. Increased intra-abdominal pressure is one of the main factors associated with a higher prevalence of GERD among individuals with obesity. The accumulation of intra-abdominal fat around the stomach increases intragastric pressure, displaces the lower esophageal sphincter and changes the astro-esophageal gradient, leading to an increased exposure of the esophagus to gastric content [5]. Furthermore, hiatal hernias (HHs) also increase the risk of GERD symptoms, and a higher prevalence of HHs is observed among individuals with obesity [6,7].

Currently, bariatric surgery is the most effective long-term treatment for obesity. The prevalence of GERD symptoms among the patients referred to bariatric surgery is in the range between 40.8% to 62.4% [8,9,10]. Given the high prevalence rate, bariatric surgeons must take GERD symptoms into consideration when choosing relevant surgical procedures. Sleeve gastrectomy (SG) is the most common primary operation for obesity in the world, but the majority of the surgeons would most likely offer Roux-en-Y gastric bypass (RYGB) for a patient with obesity and severe GERD. However, even patients with mild to moderate GERD may expect better control of their symptoms after RYGB compared to SG. In a Finnish randomized controlled study, about half of the patients with mild to moderate preoperative GERD in an SG group had their symptoms worsened compared to only less than 10% after RYGB at their 10-year follow-up [11].

Even though RYGB is the procedure of choice for patients with GERD symptoms, about one-third of them still need to take anti-reflux medications 10 years after surgery [11]. Moreover, the prevalence of GERD increases with the follow-up time. Robert M. et al. [12] have demonstrated that between 2 years and 5 years after surgery, the GERD prevalence rate increased from 1% to 18% in an RYGB group and from 6% to 41% in a one anastomosis gastric bypass (OAGB) group. The reason for this is unknown. The possible risk factors for postoperative GERD after RYGB could be insufficient weight loss or weight regain (WR), preoperative presence or development of HHs over time, larger pouch or anastomotic stricture/ulcer, eating behavior or functional gastrointestinal disorders such as irritable bowel syndrome.

So far, few studies have investigated factors associated with GERD after RYGB. Petrucciani N. et al. [10] have found that preoperative GERD, which has an impact on quality of life and anastomotic ulcers, significantly increased the odds of GERD 10 years after RYGB. Notably, lower weight loss defined as %TWL < 25 was the only protective factor [10]. The goal of this prospective study was to investigate the prevalence of GERD more than 10 years after RYGB and explore the influence of weight loss, BMI, WR, and eating and gastrointestinal functional disorders on the persistence or occurrence of GERD.

## 2. Materials and Methods

This is a continuation of our prospective study, which included 180 patients operated via RYGB from September 2010 to January 2013 at the Surgery Department, Lithuanian University of Health Sciences Hospital. The detailed protocol and one-year follow-up results were published previously [13]. In summary, patients were eligible for this study if they fulfilled standard criteria for bariatric surgery, which were approved by the Lithuanian Sickness Fund (age between 18 and 65 years, and BMI ≥ 40 kg/m^2^ or BMI ≥ 35 kg/m^2^ with at least one co-morbidity, such as hypertension, type 2 diabetes mellitus, arthrosis, and sleep apnea or infertility for woman), and signed informed consent. The ethical committee of the Lithuanian University of Health Sciences Hospital approved this study (protocol Nr. BE-2-59).

The proportion of males and females was 28.9% and 71.1%, respectively. Average age was 42.7 (10.5) years and BMI was 45.2 (6.4) kg/m^2^. In all cases, antecolic-antegastric laparoscopic RYGB with linear staple technique was performed. The gastric pouch was 20–30 mL in size, 50 cm biliopancreatic limb and 150 cm Roux limb were created, and both mesenterial windows were closed with nonabsorbable continuous suture. In the current study, patients were interviewed online on average 12.2 (0.6) years after primary surgery. Information about patients’ self-reported weight changes, GERD and gastrointestinal symptoms, and eating behavior was collected.

GERD symptoms were evaluated at baseline and 12 years after RYGB using the Gastroesophageal Reflux Disease-Health Related Quality of Life (GERD-HRQL) questionnaire [14]. GERD-HRQL was developed to estimate the typical symptoms of GERD and assess responses to medical or surgical treatments. It consists of a total of 11 items, 10 of which are measured from 0 to 5 and are used to calculate the total GERD-HRQL score by adding the individual item scores. Item 11 evaluates a patient’s satisfaction regarding their present state by asking if they are satisfied, neutral or dissatisfied. The GERD score was diagnosed preoperatively if the patients had erosive esophagitis on gastroscopy or were on continuous PPI treatment or if the GERD-HRQL score was equal or higher than 8. Postoperative GERD diagnosis was based on clinical data, such as continuous PPI treatment or a GERD-HRQL score of 8.

Gastrointestinal symptoms were evaluated by the Gastrointestinal Symptom Rating Scale (GSRS) questionnaire [15], which was initially designed to capture the symptoms present in peptic ulcer disease and irritable bowel syndrome. The questionnaire has 15 items rated on a 7-point Likert scale, where 1 represents no symptoms and 7 indicates severe symptoms. The mean values for diarrhea (represented by 3 items in the score), indigestion (4 items), constipation (3 items), abdominal pain (3 items), and reflux (2 items) were calculated. The higher the score, the more severe the symptoms.

Three different aspects of eating behavior—cognitive restraint (CR), uncontrolled eating (UE) and emotional eating (EE)—were evaluated by the TFEQ-R18 questionnaire. CR is a restriction of food intake to maintain body weight, UE is a loss of control of eating provoked by a feeling of hunger, and EE is caused by emotional stimulus. There are 18 items, and each is scored between 1 and 4 [16]. The item scores were summated into CR, UE, and EE scale scores. The raw scale scores were transformed to a 0–100 scale [100 − (((raw score − lowest possible raw score)/possible raw score range) × 100)]. The higher values on the respective scales are more indicative of the aforementioned behaviors.

Percent excess BMI loss (%EBMIL) at the 12-year follow-up was estimated with the following formula: [preoperative BMI kg/m^2^ − BMI kg/m^2^ at 12-year follow-up]/[preoperative BMI kg/m^2^ − 25 kg/m^2^] × 100. Percent weight regain (%WR) was estimated as [total body weight in kg at 12-year follow-up − total body weight in kg at nadir]/[total body weight in kg at baseline − total body weight in kg at nadir] × 100. Percent of total weight loss (%TWL) was calculated as [total body weight in kg at baseline − total body weight in kg at 12-year follow-up]/total body weight in kg at baseline × 100.

### Statistical Analysis

The SPSS program, version 29.0 (SPSS Inc., Chicago, IL, USA), was used for the statistical analyses. Normality of variables was estimated with the Kolmogorov–Smirnov test. Continuous variables with normal distribution are presented as means and standard deviations (SDs), and abnormally distributed ones are displayed as medians with an interquartile range (IR). Categorical variables are presented in numbers and percentages. The GERD-HRQL scores, TFEQ-R18 factor scores and GSRS dimension scores were abnormally distributed at baseline and at the 1-year and 12-year follow-ups. The means of these paired sample scores were compared using the Wilcoxon signed-rank test. Cut-off values of continues variables for univariate and multivariate logistic regression were identified using ROC analysis. Multivariate logistic regression was used to identify significant predictors of GERD at the 12-year follow-up. A *p* value < 0.05 was considered statistically significant.

## 3. Results

Preoperative patient characteristics are presented in Table 1. During preoperative gastroscopy, HHs were diagnosed in 37 (20.6%) cases. Forty-nine (27.2%) patients had erosive esophagitis and thirty-seven (20.6%) were on continuous PPI treatment. Finally, 74 (41.1%) patients at baseline had erosive esophagitis or daily PPI treatment or GERD-a HRQL score of 8 and were diagnosed with GERD. Twelve years after RYGB, 30 (23.8%) patients had clinically diagnosed GERD and twenty (15.9%) patients were on anti-reflux medications. Among those who had no GERD at baseline, fifty-four (75%) continued to have no GERD symptoms at the 12-year follow-up and eighteen (25%) developed de novo GERD. Among the patients who had GERD, 42 (77.8%) reported that GERD resolved and 12 (22.2%) continued to have GERD symptoms (Table 2).

At the 12-year follow-up, we contacted and collected responses from 126 (70%) patients—38 (30.2%) males and 88 (69.8%) females. The average BMI was 33.0 (5.9), and %EBMIL and %TWL were 60.37 and 25.73%, respectively. The median %WR was 18.0 (39.0), and 71 patient (59.7%) experienced > 10%WR.

The GERD-HRQL score at baseline was 3.0 (9.0) and decreased to 0 (1.0) (*p* < 0.001) and 2.0 (5.0) (*p* = 0.028) 1 year and 12 years after surgery, respectively. A significant increase (*p* < 0.001) in the GERD-HRQL score was observed between the 1-year and 12-year follow-ups (Table 3). The GSRS Diarrhea score at baseline was 1.33 (0.67), 1.0 (0.67) after 1 year, and 1.5 (2.42) after 12 years. The change between the baseline and 1-year scores was insignificant; however, 12 years after RYGB, the GSRS Diarrhea score increased significantly compared to the baseline (*p* < 0.001) and 1-year (*p* = 0.002) scores. The GSRS Indigestion score was 2.0 (1.25) at baseline. One year after RYGB, it decreased to 1.25 (0.75) (*p* < 0.001); however, 12 years after surgery, it increased to 2.25 (1.25) (*p* < 0.001). The GSRS Constipation score at baseline, 1 year, and 12 years after RYGB was 1.33 (1.0), 1.0 (1.0), and 1.0 (1.0), respectively, without significant changes. The GSRS Pain score at baseline was 1.67 (1.0) and decreased to 1.0 (0.67) (*p* < 0.001) after one year and stayed at the similar level 1.0 (0.33) even twelve years after surgery. The GSRS Reflux score was 1.0 (1.0), 1.0 (0.0), and 1.0 (1.0) at baseline, 1 year, and 12 years after surgery, respectively. Significant changes were observed between the baseline and 1-year scores (*p* < 0.001) and between the 1-year and 12-year scores (*p* < 0.001) (Table 3).

The UE score at baseline was 51.85 (22.22) and decreased after 1 and 12 years to 25.93 (7.41) (*p* < 0.001) and 40.74 (33.3) (*p* < 0.001), respectively. A statistically significant (*p* < 0.001) increase in the UE score from the 1-year to the 12-year follow-up was observed. The baseline value for CR was 52.78 (11.1), and it increased to 55.56 (11.11) (*p* = 0.007) 1 year after surgery. The 12-year CR score was 55.56 (16.67) and did not reach statistical significance compared to the baseline and 1 year follow-up scores. The EE score at baseline was 44.44 (44.44) and decreased to 11.1 (11.11) 1 year after surgery (*p* < 0.001). Twelve years after surgery, the EE score increased to 33.3 (22.2) (*p* < 0.001) but was still significantly lower compared to the baseline EE score (*p* < 0.001).

Three variables, which were significant in the univariate analysis (*p* < 0,05), were entered into the multivariate logistic regression model (Table 4). %WR > 11 (OR 3.22, 95% CI 1.13 to 9.22, *p* = 0.029) and the GSRS Diarrhoea score (OR 3.21, 95% CI 1.14 to 9.06, *p* = 0.027) were significant predictors of GERD 12 years after RYGB. Our model accounted for the 22.2% of total variance, with the correct prediction rate being 76.7%.

## 4. Discussion

This study was an extension of our previously published prospective study [13] and provided 12-year follow-up data on weight loss, GERD and gastrointestinal symptoms, and eating behavior after RYGB. More than half of our patient population experienced ≥ 10%WR, and weight regain was found to be a significant predictor of GERD 12 years after surgery. Another factor that increased the risk of GERD was the GSRS Diarrhea score (by more than 3.2). These findings allowed us to obtain some insights as to why GERD continues or appears de novo after RYGB.

Previous studies have demonstrated an increased prevalence of GERD in a range from 40.8% to 62.4% among bariatric surgery candidates [8,9,10]. Similarly, 41.1% of bariatric surgery patients presenting in our center had GERD preoperatively. RYGB is considered one of the most effective procedures in the treatment of obesity and GERD. Recently, Salminen et al. [11] published the results of a 10-year randomized controlled study comparing SG and RYGB. Patients with severe GERD and large hiatal hernias were excluded. After 10 years, 91 patients with SG and 85 patients with RYGB underwent endoscopy. Erosive esophagitis was more common after SG than after RYGB—31% vs. 7% (*p* < 0.001), respectively. Less patients after RYGB compared to SG were taking PPI—36% vs. 64% (*p* < 0.001), respectively—and only 9% of patients after RYGB compared to 49% after SG (*p* < 0.001) experienced worsening of GERD symptoms [11].

The possible mechanisms responsible for an improvement in GERD after RYGB could be related to decreased intragastric pressure, the exclusion of fundus, and the creation of a low-pressure gastric pouch [17]. Similar anatomical and physiological changes are also observed in another gastric bypass modification—OAGB. However, 5-year follow-up results of a randomized controlled trial comparing RYGB and OAGB have shown that the prevalence of GERD was lower after RYGB—18% and 41%, respectively [12]. In contrast, Hany M. et al. [18] did not find any difference in the prevalence of GERD at a 2-year follow-up among patients who underwent RYGB and OAGB as a revisional procedure after failed SG. They included 80 patients in each group, and none of the patients after OAGB and one (1.3%) patient after RYGB had erosive esophagitis. Importantly, about one-third of the patients in each group underwent HH repair during revisional surgery [18]; this additional procedure could have reduced the prevalence of GERD after both procedures. However, further studies with long-term follow-ups are needed to address the resolution of GERD symptoms and the occurrence of de novo GERD rates after OAGB.

Even though RYGB effectively controls GERD, some patients experience the persistence or onset of GERD symptoms. In the present study, 12 years after RYGB, the resolution of GERD was observed in 77.8% of cases, with 25% reporting de novo GERD. Similarly, Santanicola A. et al. [9] investigated 45 patients on average 99.9 months after RYGB and found a GERD resolution rate of 69.6% and new onset of GERD in 18.2% of cases. The risk factors associated with postoperative GERD after RYGB are poorly understood. Petrucciani N. et al. [10] have found that preoperative GERD (OR 2.65, *p* < 0.0001) with glycemic imbalances defined as post-prandial symptoms, which occur more than 2 times per month and have an impact on patients’ quality of life (OR 2.42, *p* = 0.006), anastomotic ulcer occurrence (OR 5.26, *p* < 0.0001), and %TWL at 10 years < 25 (OR 0.52, *p* = 0.021), were significant risk factors associated with GERD 10 years after RYGB [10]. We were unable to confirm the finding that preoperative GERD has an influence on postoperative GERD because more patients in our study developed de novo GERD compared to those who had persistent GERD. We asked our patients about current diseases, and no one mentioned anastomotic ulcers. However, we had no data about the history of peptic ulcers; thus, we were unable to include anastomotic ulcers as a possible risk factor into our analysis.

In our study, BMI, %EBMIL, and %TWL at the 12-year follow-up had no impact on the development of postoperative GERD. However, a WR value of more than 11% was a significant risk factor (OR 3.22, *p* = 0.029) associated with postoperative GERD. Increased intra-abdominal pressure may explain such a finding, but the diet or eating behavior of a patient may also have an impact on the persistence or occurrence of GERD symptoms. The Nurses’ Health Study II included 42,955 women and identified 9291 cases with GERD symptoms with a follow-up of 392,215 person-years. A higher prudent dietary score and ≤2 cups daily of coffee, tea, or soda reduced the risk of GERD symptoms, with their hazard ratios being 0.87 (0.84–0.91) and 0.92 (0.88–0.97), respectively [19]. A recent study in a Chinese student population found that emotional eating was related to the laryngopharyngeal reflux (OR 6.8, *p* < 0.001) [20]. However, in our study, there was no significant correlation between emotional eating, uncontrolled eating, or cognitive restraint and the GERD-HRQL score at baseline and 1 year after RYGB [13]; moreover, after 12 years, there was no evident relation between eating behavior and postoperative GERD.

In the present study, HH among the patients undergoing bariatric surgery was diagnosed in 20.6% of cases and was comparable to the results of the recent studies, where the prevalence of HH in similar populations was in a range from 15.7% to 47.8% [8,9]. The majority of bariatric surgery patients have small HH, with HH larger than 2 cm consisting only in 10% of the cases [21]. Larger HHs are associated with higher % proximal acid exposure time, acidic refluxes, and reflux episodes detected by impedance. Thus, it is reasonable to perform HH repair when the size of HH is >2 cm. There is still controversy regarding the role of small HHs on the persistence of GERD after RYGB. Khouri A. et al. [22] compared GERD-HRQL scores 1 year after RYGB between patients who had small HHs without repair and patients without HHs. In both groups, the GERD-HRQL score decreased significantly from baseline to 1 year, and there was no significant difference in the GERD-HRQL score between the groups 1 year after surgery [22]. In our patient cohort, no HH repairs were carried out during the primary procedure, and 12 years after surgery, the presence of HH on preoperative endoscopy did not increase the risk of persistent or de novo GERD. However, without postoperative endoscopy, we were unable to find out how many patients had an increase in the size of their HHs or developed new HHs during the 12-year period and how such changes have influenced the occurrence of de novo GERD. The recent Multi-Ethnic Study of Atherosclerosis (MESA) [23], which investigated the findings of full-lung CT cans in 3200 subjects aged 53–94 years, has demonstrated a 9 per 1000 person-years incidence rate of HHs after a 10-year follow-up among 1464 participants free of HHs at baseline. Furthermore, among the 75 participants with HHs and at the 10-year follow-up, the median HH area increased from 9.9 cm^2^ to 17.9 cm^2^ (*p* = 0.02) [23].

We have also explored the association between gastrointestinal symptoms and GERD 12 years after surgery. Only the GSRS Diarrhea score of more than 3.2 was significantly related to postoperative GERD (OR 3.21, *p* = 0.027). A systematic literature review has shown significant overlap between irritable bowel syndrome (IBS) and GERD, where the prevalence of GERD among the patients diagnosed with IBS was 39.3% and IBS prevalence among patients with GERD reached 48.8% [24]. One explanation for this could be that GERD and IBS symptoms are caused by similar underlying gastrointestinal dysfunctions [25]. However, despite a significant increase in the GSRS Diarrhea score among our patients from baseline to 12 years after RYGB, the GSRS Pain score decreased significantly 1 year after surgery and remained at a similar level even 12 years later. Bothersome abdominal pain symptoms (GSRS Pain score ≥ 3) were observed in 15.1% of patients preoperatively and only in 5.1% and 4.0% of cases after 1 and 12 years, respectively. Given that the main symptom of IBS is abdominal pain, it is likely that our patients had functional diarrhea more often than diarrhea caused by IBS. Furthermore, other functional disorders such as esophageal or Roux limb dysmotility have been observed after RYGB or total gastrectomy with Roux-en-Y reconstruction [26,27], suggesting that esophageal or Roux-en-Y limb functional disorders may play a greater role in the persistence or occurrence of GERD symptoms after RYGB than IBS.

This study has several limitations. First, we used self-reported weight to estimate long-term weight loss and weight regain. It is known that patients with obesity tend to under-report their actual weight. However, we assumed that under-reporting did not influence the results of the present study because even a relatively small %WR was significantly related to postoperative GERD. Second, the rate of hiatal hernias in our population could be underestimated because preoperative gastroscopy was carried out in different centers without a uniform protocol and was not supplemented by an upper GI study, which is more sensitive in diagnosing hiatal hernias [28]. Third, there were no HH repairs performed in our patient cohort, suggesting that all HHs were small; other studies have demonstrated an average 10% prevalence rate of larger HHs among bariatric surgery patients [21]. Thus, the finding that the preoperative presence of HHs had no impact on postoperative GERD can be attributed only to the patient population that underwent bariatric surgery with small HHs. Finally, the 12-year GERD diagnosis was based on clinical data, and postoperative gastroscopy was not routinely performed. Thus, some cases of silent GERD could have been missed, leading to the underestimation of the prevalence of GERD after RYGB.

## 5. Conclusions

RYGB is an effective procedure to control GERD; however, 23.8% of our patients had persistent or de novo GERD after 12 years. The independent risk factors associated with GERD after RYGB were a WR prevalence of > 11% and a GSRS Diarrhea score of > 3.2. Future research is needed to better understand the mechanisms that lead to the persistence or onset of GERD after RYGB.

## Figures and Tables

**Table 1:** Preoperative patient characteristics.

Characteristics	Patients (*n* = 180)
Age, y mean (SD)	42.7 (10.5)
Sex, M/F	52/128
BMI, kg/m^2^ mean (SD)	45.2 (6.4)
Waist circumference, cm mean (SD)	127.1 (15.6)
Smoking *n* (%)	59 (32.7)
Erosive esophagitis *n* (%)	49 (27.2)
Hiatal hernia *n* (%)	37 (20.6)
GERD-HRQL score, median (min/max)	3.0 (8.0)
PPI use *n* (%)	37 (20.6)
GERD *n* (%)	74 (41.1)
Hypertension *n* (%)	108 (60)
Diabetes mellitus *n* (%)	14 (7.8)
Dyslipidemia *n* (%)	131 (72.8)

**Table 2:** GERD before and 12 years after RYGB.

GERD before Surgery	GERD after Surgery		*p* = 0.717
	Yes	No	Total
Yes	12	42	54
No	18	54	72
Total	30	96	126

**Table 3:** GERD-HRQL, GSRS, and TFEQ-R18 results at baseline, 1 year, and 12 years after RYGB.

	Baseline *n* = 179	1 Year *n* = 97	12 Years n = 126	*p* Value B vs. 1 y	*p* Value B vs. 12 y	*p* Value 1 y vs. 12 y
GERD-HRQL score				<0.001	0.028	<0.001
Median (IR)	3.0 (9.0)	0.0 (1.0)	2.0 (5.0)			
Mean (SD)	5.07 (5.21)	0.98 (2.52)	3.94 (4.77)			
GSRS Diarrhea score				0.143	<0.001	0.002
Median (IR)	1.33 (0.67)	1.0 (0.67)	1.5 (2.42)			
Mean (SD)	1.63 (0.84)	1.46 (0.84)	2.37 (1.78)			
GSRS Indigestion score				<0.001	0.398	<0.001
Median (IR)	2.0 (1.25)	1.25 (0.75)	2.25 (1.25)			
Mean (SD)	2.29 (0.97)	1.45 (0.59)	2.40 (0.80)			
GSRS Constipation score				0.433	0.204	0.917
Median (IR)	1.33 (1.33)	1.17 (1.0)	1.0 (1.0)			
Mean (SD)	1.81 (0.99)	1.53 (0.75)	1.67 (1.19)			
GSRS Pain score				<0.001	<0.001	0.713
Median (IR)	1.67 (1.0)	1.0 (0.67)	1.0 (0.33)			
Mean (SD)	1.85 (0.80)	1.38 (0.61)	1.32 (0.61)			
GSRS Reflux score				<0.001	0.162	<0.001
Median (IR)	1.0 (1.0)	1.0 (0.0)	1.0 (1.0)			
Mean (SD)	1.62 (0.90)	1.09 (0.29)	1.48 (0.95)			
Uncontrolled Eating score				<0.001	<0.001	<0.001
Median (IR)	51.85 (22.22)	25.93 (7.41)	40.74 (33.33)			
Mean (SD)	50.99 (15.58)	29.63 (9.93)	38.83 (18.48)			
Cognitive Restraint score				0.007	0.339	0.107
Median (IR)	52.78 (11.11)	55.56 (11.11)	55.56 (16.67)			
Mean (SD)	51.94 (10.12)	55.44 (8.14)	53.53 (11.34)			
Emotional Eating score				<0.001	<0.001	<0.001
Median (IR)	44.44 (44.44)	11.11 (11.11)	33.33 (22.22)			
Mean (SD)	46.93 (28.74)	20.14 (22.01)	31.22 (14.38)			

**Table 4:** Factors associated with GERD 12 years after RYGB.

Factor	Univariate Model			Multivariate Model	
	N	Beta (95% CI)	*p* Value	Beta (95% CI)	*p* Value
Sex (Ref = male)	126	1.25 (0.50, 3.13)	0.633		
Age, years (Ref ≤ 55.6)	126	1.28 (0.56, 2.92)	0.559		
BMI kg/m^2^ (Ref ≤ 31.7)	120	1.88 (0.78, 4.51)	0.157		
%EBMIL (Ref > 39.9)	120	0.79 (0.24, 2.60)	0.700		
%TWL (Ref > 18.7)	120	0.78 (0.26, 2.32)	0.658		
%Weight regain (Ref ≤ 11.0)	120	3.22 (1.19, 8.67)	0.021	3.22 (1.13, 9.22)	0.029
Uncontrolled eating (Ref ≤ 47.0)	126	2.00 (0.87, 4.60)	0.103		
Cognitive restraint (Ref ≤ 41.7)	126	1.50 (0.47, 4.84)	0.497		
Emotional eating (Ref ≤ 38.9)	126	0.82 (0.31, 2.14)	0.684		
GSRS Diarrhea score (Ref ≤ 3.20)	126	5.31 (2.19, 12.91)	<0.001	3.21 (1.14, 9.06)	0.027
GSRS Indigestion score (Ref ≤ 2.15)	126	4.73 (1.77, 12.60)	0.002	2.45 (0.81, 7.42)	0.113
GSRS Constipation score (Ref ≤ 1.5)	126	0.86 (0.35, 2.01)	0.734		
Preoperative GERD (Ref = no)	126	1.17 (0.51, 2.69)	0.717		
Hiatal hernia, (Ref = no)	106	0.62 (0.16, 2.34)	0.475		

## Data Availability

The original contributions presented in the study are included in the article, further inquiries can be directed to the corresponding author.
